# Tough Hydrogels Based on Maleic Anhydride, Bulk Properties Study and Microfiber Formation by Electrospinning

**DOI:** 10.3390/polym13060972

**Published:** 2021-03-22

**Authors:** Faiza Bettahar, Fadila Bekkar, Leyre Pérez-Álvarez, Mohammed Issam Ferahi, Rachid Meghabar, José Luis Vilas-Vilela, Leire Ruiz-Rubio

**Affiliations:** 1 Laboratoire de Chimie des Polymères, Université Oran1 Ahmed Ben Bella, El-Mnao uer, BP 1524, Oran 31000, Algeria; faiza-bettahar@hotmail.com (F.B.); bekkar31@outlook.com (F.B.); mohammed.ferrahi@yahoo.fr (M.I.F.); rachidmeghabar@yahoo.fr (R.M.); joseluis.vilas@ehu.eus (J.L.V.-V.); 2 Macromolecular Chemistry Group (LQM), Physical Chemistry Department, Faculty of Science and Technology, University of the Basque Country (UPV/EHU), 48940 Leioa, Spain; leyre.perez@ehu.eus; 3 BCMaterials, Basque Center for Materials, Applications and Nanostructures, UPV/EHU Science Park, 48940 Leioa, Spain

**Keywords:** hydrogel nanofiber, electrospinning, maleic anhydride, tough hydrogels

## Abstract

Hydrogels present a great number of advantages, such as their swelling capacity or their capability to mimic tissues, which make them very interesting biomaterials. However, one of their main disadvantages is their lack of good mechanical properties, which could limit some of their applications. Several strategies have been carried out to develop hydrogels with enhanced mechanical properties, but many of the suggested synthetic pathways to improve this property are expensive and time consuming. In this work, we studied an easy synthetic path to produce tough hydrogels based on different maleic anhydride copolymers crosslinked with polyethylenglycol. The effect of the comonomers in the mechanical properties has been studied, their excellent mechanical properties, good swelling behavior and thermal stability being remarkable. In addition, in order to evaluate their possible applications as scaffolds or in wound healing applications, microsized fibers have been fabricated by electrospinning.

## 1. Introduction

Among the polymeric materials, hydrogels are one of the most widely studied and used biomaterials due to their versatility and swelling capability, in addition to their tailorable structures and properties that make them very attractive candidates for many applications, such as drug delivery, tissue engineering and wound dressing [1,2,3,4]. However, among the main drawbacks of this kind of materials is their poor mechanical properties, present in both synthetic and natural hydrogels. Indeed, the synthesis of new hydrogels or the modification of the existing ones to improve their mechanical properties have become an important study field in recent years [5,6]. Until now, several strategies have been described for the development of strong mechanical hydrogels, the main ones including: composite hydrogels [7,8] and double network hydrogels [9,10]. Nonetheless, the synthesis of mechanical tough hydrogels often requires complex synthetic pathways and it could be considered expensive and time consuming, reducing the potential application of these materials [11]. In this work, hydrogels with excellent compression properties have been developed based on the crosslinking of different copolymers of maleic anhydride by an esterification reaction with polyethylene glycol (PEG).

As mentioned above, maleic anhydride has been used as the main material for the development of hydrogels. Several maleic anhydride-based materials have been reported to date, many of them being used as adhesives, biomedical devices and drug delivery systems [12,13,14]. As an example, Singh et al. [15] analyzed the influence of poly(ethylene glycol) (PEG) on the physicochemical properties of the hydrogels prepared from Gantrez^®^ AN-139, a copolymer of methyl vinyl ether and maleic anhydride. The authors described tailored drug delivery by varying the crosslinking of the hydrogels by the incorporation of PEG with different molecular weights. Similarly, Caló et al. [16] reported a Gantrez^®^ and poly(vinyl alcohol)-based hydrogel, which presented good antibacterial and mechanical properties. These properties added to their good adhesion to porcine skin, making them highly suitable for wound dressing applications.

It is also important to highlight its biocompatibility and low cytotoxicity that makes the maleic anhydride derivatives excellent biomaterials [17,18]. One interesting approach for wound dressing applications is based on nanofiber mats that present high porosity and a large surface area. These properties allow the proliferation and migration of cells involved in the wound healing process [19]. One of the most used methodologies to develop this kind of structure is electrospinning, due to its cost-effectiveness compared to other time-consuming and expensive techniques employed in the development of highly porous polymeric structures [20,21]. As an example, Yang et al. [22] fabricated a gelatin/dextran–maleic anhydride-based hydrogel by electrospinning. In this study, the polymer blend fibers were formed by electrospinning, and then crosslinked by photocrosslinking reaction. These materials presented a good biocompatibility and the in vitro studies showed that they were able to support cell proliferation and adhesion, making them highly suitable materials for tissue engineering and wound dressing applications. In addition to wound healing applications, these fiber mats could be used as drug delivery systems. Varshosaz et al. [23] reported the preparation of nanofiber mats by poly(methyl vinyl ether-co-maleic acid) as a drug delivery system exploiting the large surface area as a fast dissolving carrier.

The aim of this work was the synthesis of maleic anhydride-based hydrogels with high mechanical strength, and good swelling capability by an easy synthetic procedure. This synthetic path could be considered as an efficient procedure, compared to other time-consuming process such as double network fabrication commonly employed in the development of tough hydrogels. In addition, this highly versatile process was employed for the fabrication of micrometric-sized anhydride maleic-based hydrogel fibers by electrospinning. It is important to notice that the crosslinking process was successfully carried out without losing their mat structure and surface area after crosslinking as it is often described for those systems in which the crosslinking is performed after the fibers formation. The excellent mechanical properties of the maleic anhydride-based hydrogels obtained in this study added to their good swelling capacity and their fiber formation capability make them excellent candidates for several biomedical applications such as wound healing, tissue engineering and drug delivery.

## 2. Experimental

### 2.1. Materials

Poly(ethylene glycol) (PEG, Mn = 200 g/mol), poly(ethylene-alt-maleic anhydride) (Et-MA, Mw = 10,000–500,000 g/mol), poly(isobutylene-alt-maleic anhydride) iBu-MA (Mw = 6000–12,200 g/mol), poly(methyl vinyl ether-alt-maleic anhydride) (MVE-MA, Mw = 216,000 g/mol) and acetone were obtained from Sigma-Aldrich (Darmstadt, Germany), *N*,*N*-dimethyl formamide (DMF) from Macron Fine Chemicals (Deventer, The Netherlands).

### 2.2. Synthesis of the Hydrogels

In order to synthetize the hydrogels, 35% wt. of each copolymers and 10% wt. of polyethylene glycol, as a crosslinker, were solved in (2:1) (*v*/*v*) acetone/DMF mixture. The mixtures were places in a round mold and allowed to react for 3 days.

### 2.3. Hydrogel Fibers Fabrication by Electrospinning

Hydrogel nanofibers were prepared using the electrospinning method. Copolymer solutions (30% *w*/*v* of the corresponding copolymer mixed with 10% (*w*/*w*) (weight percent to copolymers) of PEG in acetone/DMF mixture 2:1 *v*/*v*) were prepared and loaded in a 30 mL plastic syringe. The syringe with a 0.7 mm inner diameter needle (gauge 20) was attached to the pump (200 series, KD Scientific Inc., Holliston, MA, USA). The parameters used for the electrospinning of these materials were previously optimized parameters for these systems, the distance to the aluminum foil collector being fixed at 27 cm, with a voltage of 18 KV and flow rate 0.002 mL h^−1^. Copolymer nanofibers membranes were put in an oven at 100 °C for 10 min to complete the crosslinking reaction and dried at 50 °C under vacuum to remove residual solvent.

### 2.4. Materials Characterization

#### 2.4.1. Fourier-Transform Infrared Spectroscopy (FTIR)

Nicolet Nexus FTIR (Thermo Scientific, Loughborough, UK) spectrometer analysis was used to evaluate the crosslinking of the hydrogels. All the experiments were carried out by KBr pellets, at a resolution of 4 cm^−1^ and 32 scans per spectrum.

##### Thermogravimetric Analyses (TGA)

The thermal stability of the hydrogels was analyzed using a DTG-60 Shimadzu Thermobalance (Kyoto, Japan). The samples (around 10 mg) were heated in an alumina pan from room temperature to 700 °C at 10 °C·min^−1^, and all the measurements were carried out under nitrogen atmosphere (20 mL/min). The initial degradation temperature, T_i_, was determined from the intersection between the tangent to the baseline and the inflection point in the thermogram.

#### 2.4.2. Compressive Stress/Strain Study

The compressive strength of the hydrogels was measured by universal testing machine (Ibertest, Madrid, Spain), equipped with a 10 kN load cell. Five samples of each hydrogel were prepared in cylinder form (diameter 25 mm and height 10 mm) and placed in parallel plates with a distance between them equal to the height of the hydrogel. The test strain rate was of 50 mm/min. The Young’s modulus was calculated between 10–20% compressive strains in the fixed displacement mode. Each measurement was repeated three times.

#### 2.4.3. Viscosity Measurements

The viscosity is an important parameter for the electrospinning. The viscosity of the different samples was measured by a Brookfield programmable DV2-T (Brookfield Inc., Middleboro, MA, USA) viscometer at room temperature.

#### 2.4.4. Swelling Degree

The swelling experiments were performed by the immersion in distilled water of previously dried hydrogels at room temperature and weighting over time. The swelling degree (*w*/*w*) was calculated according to the Equation (1), where M_s_ is the mass of the swelled hydrogel at time t and M_d_ the mass of the dry hydrogel. (N = 3 for each data point):

(1) $$Swelling\;degree=\frac{M_{s}-M_{d}}{M_{d}}\times \;100$$

#### 2.4.5. Morphology of Hydrogel Nanofibers

The morphology was observed by scanning electron microscopy SEM (S-4800), with an acceleration voltage of 5 kV. The pore size of hydrogels and elecrospun hydrogels nanofibers was determined by image analyzer Fiji.

## 3. Result and Discussion

### 3.1. Maleic Anhydride-Based Hydrogels

**Figure 1:**
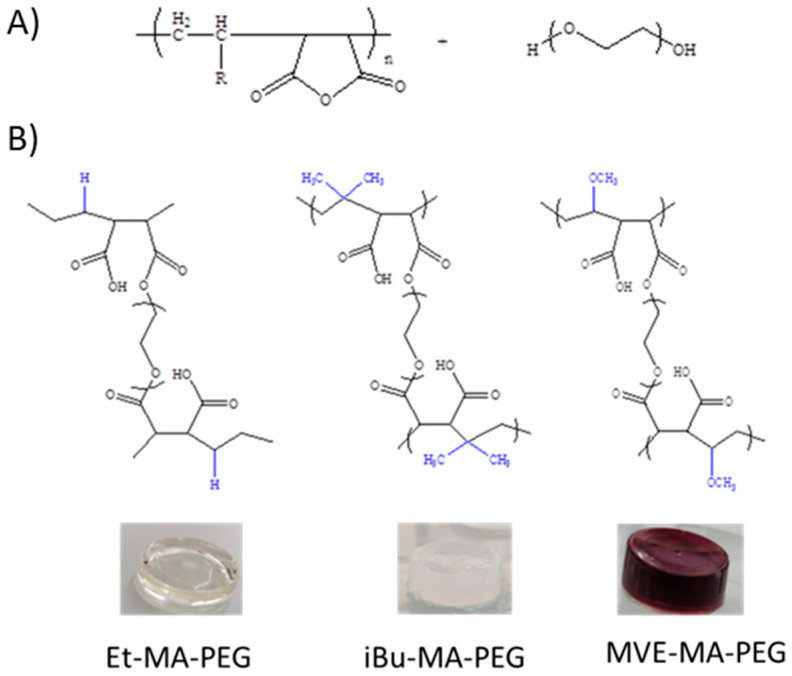
(**A**) Scheme of the general synthetic pathway for hydrogel formation; (**B**) chemical structures of the hydrogels prepared by maleic anhydride-based copolymers crosslinking with PEG.

Three different maleic anhydride-based copolymers were used for the hydrogel synthesis. The selected alternating copolymers present comonomers of different natures or size, which allow the study of the influence of the comonomer in the properties of the final hydrogels. These comonomers were ethylene, isobutylene and methyl vinyl ether. The hydrogels were obtained by the opening of maleic anhydride ring and the subsequent esterification of the carboxylic acid with the hydroxyl groups of PEG molecules (Figure 1). The magenta color of the MVE-MA-PEG hydrogel is related to the DMF solvent, similarly to that observed by Zhao et al. [24], who described the solvatochromic properties of the poly(maleic anhydride-*alt*-vinyl acetate)].

**Figure 2:**
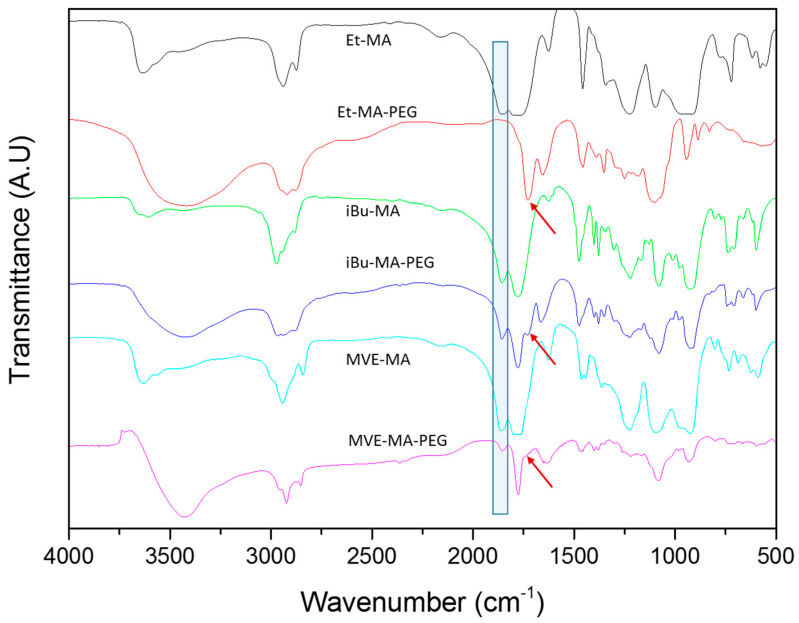
FTIR spectra of the pristine copolymers and prepared hydrogels.

Once the hydrogels were formed, their chemical structure was evaluated by FTIR spectroscopy. The Figure 2 shows the FTIR spectra of the copolymers and the obtained hydrogels. The copolymers presented typical anhydride group bands around 1770–1850 cm^−1^, ascribed to C=O symmetric and asymmetric stretching, respectively [25]. After crosslinking, most of the anhydride groups reacted during esterification or remained as free carboxylic groups after being partially hydrolyzed [26,27]. The anhydride ring opening and the carboxylic acid formation could be observed with the disappearance of the band around 1850 cm^−1^ for all the copolymers (blue rectangle at Figure 2). As could be observed, there was a shift in the characteristic peaks due to the carboxylic acid formation and the subsequent esterification reaction with the reaction with the hydroxyl groups present on the PEG. The symmetric C=O band slightly shifted due to the free carboxylic acid formation around 1780 cm^−1^. However, this band was still close to the asymmetric C=O band of the anhydride. On the other hand, the esterification reaction between the carboxylic acid groups derived from the ring opening and the hydroxyls groups of the PEG were confirmed by the presence of the ester C=O stretching band at 1730 cm^−1^ (red arrows at Figure 2) [25,26,27]. In addition, the presence of free carboxylic acids of the hydrogels and free hydroxyl of PEG is confirmed by a broad band of the OH stretching vibration around 3500 cm^−1^.

Finally, it could be noticed that the ester band observed at 1730 cm^−1^ presented significant intensity variations depending on the copolymer (red arrows at Figure 2). The Et-MA-PEG hydrogel presented a clear C=O ester peak, while, for the iBU-MA-PEG and MVE-MA-PEG hydrogels, the band ascribed to ester could be observed as a shoulder at 1730 cm^−1^. This divergence could be related to the steric hindrance of the comonomer that could complicate the esterification reaction.

### 3.2. Swelling Ratio of Hydrogels

**Figure 3:**
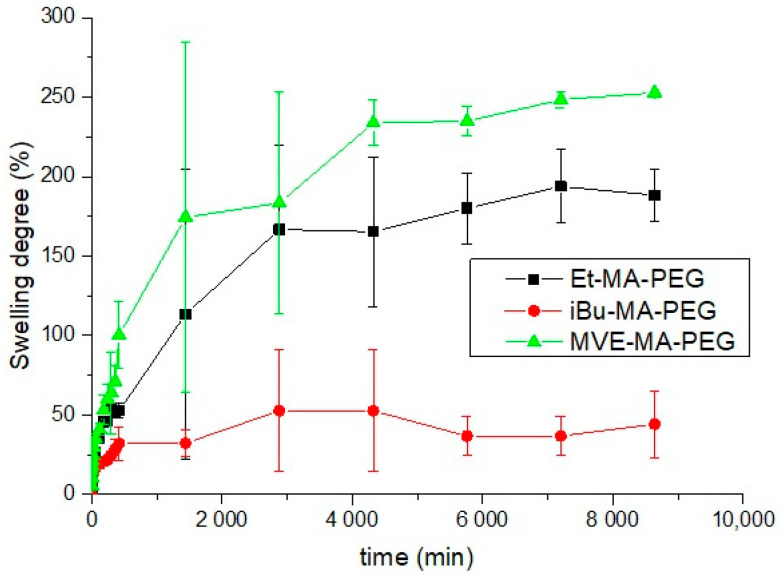
Swelling degree of the copolymers hydrogels in water.

The swelling capability could be a very useful property for many of potential applications such as drug delivery, among others. In Figure 3, the swelling degree of the hydrogels formed with the different maleic anhydride copolymers are depictured. As it can be observed, the maximum swelling was observed for MVE-MA-PEG hydrogel with a swelling equilibrium of 250%, which is consistent with the swelling results described by Singh et al. [15] for different formulations of this system. On the other hand, both ethylene and isobutylene comonomers are alkyl monomer with high hydrophobicity, so their presence could reduce the water uptake capacity of their hydrogels as it could be observed in Figure 3. The higher mobility and lower steric hindrance of the Et-MA-PEG could induce its higher swelling, maximum swelling of 150%, compared to iBu-MA-PEG. That is, the –CH_2_–CH_2_– structure of the ethylene eases the mobility of the net and enhances its swelling compared to the iBu-MA-PEG. On the other hand, the iBu comonomer presents lower mobility and higher hydrophobicity, which reduces the swelling capacity of the hydrogel, being the swelling equilibrium for iBu-MA-PEG hydrogels, 45%, the lowest of this study. This swelling behavior was similar to the one observed by other authors for polystyrene-maleic anhydride-based hydrogel (around 40%), which similarly to the iBu, presents a highly hydrophobic and voluminous comonomer [2].

### 3.3. Thermal Stability of Hydrogels

**Figure 4:**
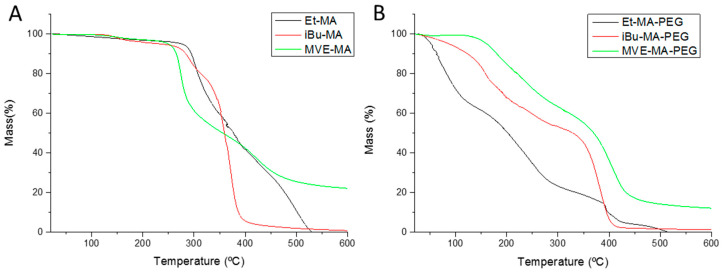
Thermal stability of the copolymer (**A**) and hydrogels (**B**).

The thermal stability of copolymers and hydrogels was evaluated by thermogravimetric analysis (Figure 4). The TGA thermograms of the copolymers (Figure 4A) showed very similar degradation paths for all copolymers, being the T_i_ of Et-MA, iBu-MA and MVE-MA at 290, 270 and 260 °C, respectively. Several authors relate the first degradation step of the copolymers with the degradation of the maleic anhydride monomer, a step which may vary slightly depending on the comonomer [28,29]. The second degradation step could be assigned to the comonomer degradation, the less the ones presenting voluminous side groups, iBu and MVE. The similarity between the copolymers with the higher steric hindrance could be related to the scission of the voluminous groups during the degradation process [30].

On the other hand, the thermal degradation of the hydrogels significantly changes due to the incorporation of the PEG and their higher water affinity (Figure 4B). In the case of Et-MA–PEG, even if the samples where dried before the TGA, water molecules remain inside the material, being exhausted in the first degradation step from 25 to 110 °C. In addition, during the hydrogel synthesis, carboxylic groups are formed due to the anhydride ring opening, subsequently reacting some of these groups with PEG in the esterification process, while others remain in the polymer chain without reacting. Considering this, the first degradation step for iBU-MA-PEG and MVE-MA-PEG, and the second for the Et-MA–PEG could correspond to the –COOH degradation which, usually, is located around 180 °C for similar polymers such as poly(acrylic acid) or poly(methacrylic acid) [31,32]. In this case, the T_i_ for this step is 177, 140, 155 °C for Et-MA–PEG, iBu-M-PEG and MVE-MA-PEG, respectively. The last degradation step, similarly to the copolymers (Figure 4A), could be ascribed to the degradation of the comonomers (Et, iBu and MVE).

### 3.4. Mechanical Properties of Hydrogels

**Figure 5:**
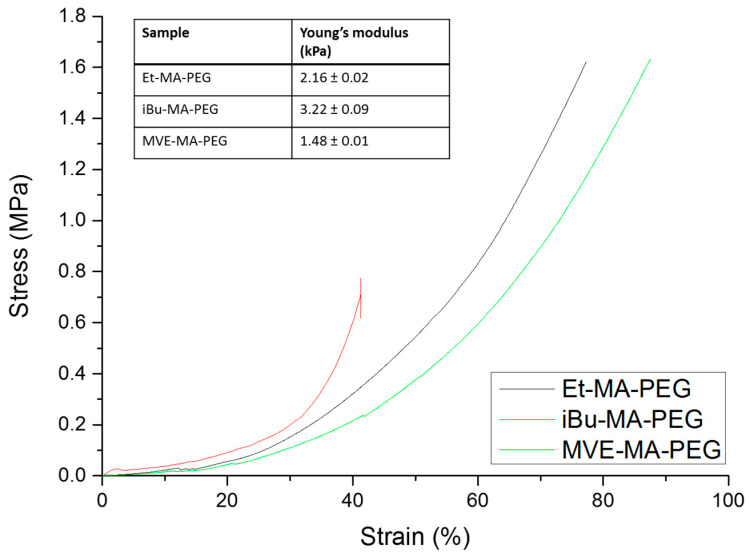
Compression stress/strain test for the synthesized hydrogels: Et-MA–PEG; iBu-MA–PEG; and MVE-MA–PEG.

Compression test of the freshly prepared hydrogels were performed by using universal testing machine, and the resultant compressive strain–stress curves are depictured at Figure 5. The hydrogel with most steric hindrance, iBu-MA-PEG, presents the lower deformation of the studied systems, breaking at 40% and 0.79 MPa, that could be associated with less mobility of the comonomer that make it more brittle. On the other hand, the compressive stress increases significantly in the other two systems, being the strain at break of 74% and 95% for Et-MA-PEG and MVE-MA-PEG, respectively, and the stress of 1.61 MPa for both of them, reaching the maximum of the loading cell. These results are correlated with the swelling capacity of these hydrogels, so it could be considered that the chain mobility of these hydrogel not only eases the water uptake, but also improves their mechanical properties. Considering the maximum stress obtained for hydrogels with the Et and MVE comonomers, these hydrogels with enhanced mechanical properties could be considered close to tough hydrogels. Usually, this toughness commonly achieved in double network hydrogels, designed for increasing the mechanical properties of the hydrogels, but at the same time they present a restricted swelling capacity that could reduce their usability in biomedical applications [33,34]. It is important to notice that soft tissues (cartilage, tendons…) present a high-water content and fracture resistance; for example, cartilage exhibits a nominal compressive modulus of 0.1–1.0 MPa [35].

### 3.5. Maleic Anhydride-Based Hydrogel Fibers

In order to study the potential application of these materials for wound healing and/or tissue engineering, micrometric size fiber mats were obtained by using electrospinning [36,37]. It is important to highlight that some studies have reported good cytotoxicity and biocompatibility for some of these copolymers, so the potential usability of hydrogels based on these copolymers could be an important alternative [38,39]. The mats were obtained by using the electrospinning technique, once the process was optimized the most adequate conditions for these materials were: needle of gauge 20 (0.7 mm inner diameter), distance to the collector of 27 cm, voltage of 18 KV and a flow rate of 0.002 mL h^−1^.

The viscosity of the copolymers at 30 *w*/*v*% was 3053 ± 307.3 cP, 26,530 ± 6062 cP, 1328 ± 852.9 cP, for the Et-MA, iBu-MA and MVE-MA, respectively. In addition, the viscosity of the formulations with PEG, previously to gel formation, was also measured, 980.8 ± 5.15 cP, 43,670 ± 6.54 cP, and 187.5 ± 4.52 cP for Et-MA/PEG, iBu-MA/PEG and MVE-MA/PEG mixtures, respectively. As could be observed, the incorporation of PEG has a different influence on the viscosity, while in Et and MVE, the viscosity decreases, in the case of the higher hindrance copolymer the viscosity increases. This effect could be ascribed to the interaction between the copolymers and the PEG when the esterification process begins, which induces an increase in the density within the molecular coil that could result in a smaller hydrodynamic volume and viscosity. This effect was also described by other authors during an esterification process to form another hydrogel [40].

In this study, first, the fiber mats of pure copolymers were fabricated to comparatively evaluate their structure with the hydrogel fibers. The hydrogel fibers were obtained by electrospinning the copolymer/PEG mixtures and their subsequent heating at 140 °C for 10 min in order to promote the complete crosslinking process. The success of this process was corroborated by FTIR.

**Figure 6:**
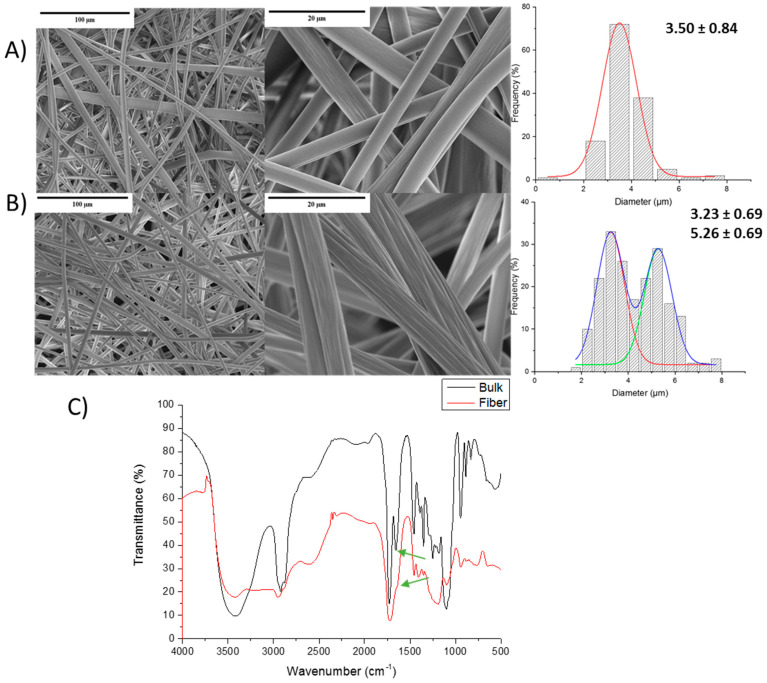
SEM images and fiber diameter of (**A**) the Et-MA copolymer; and (**B**) the Et-MA-PEG hydrogel. (**C**) FTIR spectra of bulk and fiber hydrogels.

In Figure 6, SEM images of Et-MA and its hydrogel, and the fibers’ size are depictured. Micrometric size and well-defined fibers were obtained for both systems. However, the analysis of the fibers’ diameter showed that homogeneous size fibers are present for the pure copolymer (3.50 ± 0.84 µm), whereas in the hydrogels, the morphology changes slightly, leading to two different fiber diameters: 3.23 ± 0.69 µm and 5.26 ± 0.69 µm. In addition, Figure 6C shows the FITR spectra of the bulk Et-MA-PEG hydrogel and the hydrogel obtained after the electrospinning process. As it could be observed, the same characteristic peaks are present in both spectra, confirming the hydrogel formation. In this case, the C=O peak of the ester at 1730 cm^−1^ (green arrows, Figure 6C) corresponding to the crosslinking process could be observed as a shoulder in the fiber spectra, confirming the crosslinking with PEG. However, this process could be less efficient compared to the bulk process, since bulk hydrogel presents a clear peak 1730 cm^−1^, so the number of chain segments involved in the esterification seems to be higher than that in fibers. It is important to notice that the hydrogel has formed without modifying the fibers’ morphology. In a similar system, starch/Et-MA was fabricated by Oktay et al. [41] and in this case, when the hydrogel was formed, the mat fibers merged, losing part of their surface area and reducing their potential application.

**Figure 7:**
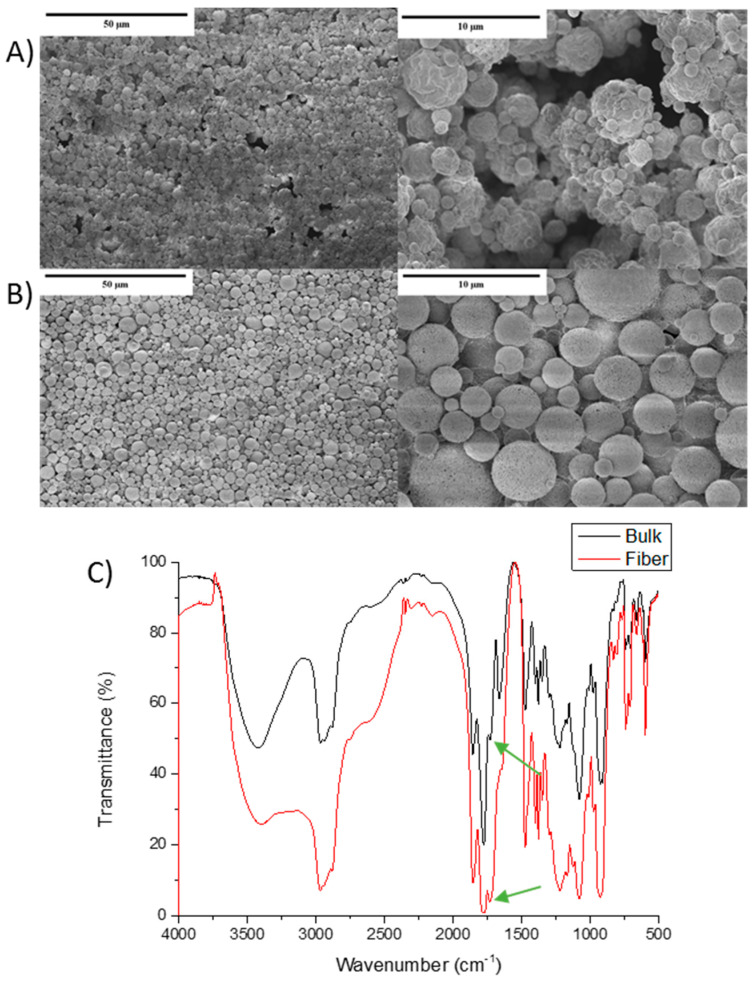
SEM images and fiber diameter of (**A**) the iBu-MA copolymer; and (**B**) the iBu-MA-PEG electrospun hydrogel (nanoparticles); and (**C**) the FTIR spectra of bulk hydrogel and hydrogel particles.

On the other hand, in accordance with the results reported in previous experiments, iBu-MA-PEG presented a significantly different behavior. In this case, the steric hindrance and the hydrophobicity of the isobutylene groups seems to prevent the formation of fibers by this technique. As could be observed in Figure 7, nano- and microparticles of copolymer (A) and hydrogel (B) were obtained from this material. It could be noticed that hydrogel nanoparticles (Figure 7B) presented a more homogeneous surface than its corresponding copolymer, an effect which could be related to the crosslinking process that could improve the particle shape of the hydrogel. Nevertheless, even if fibers were not obtained, the hydrogel nanoparticles were successfully formed, confirmed the characteristic ester formation (green arrows) being confirmed at 1730 cm^−1^ in the FTIR spectra (Figure 7C).

**Figure 8:**
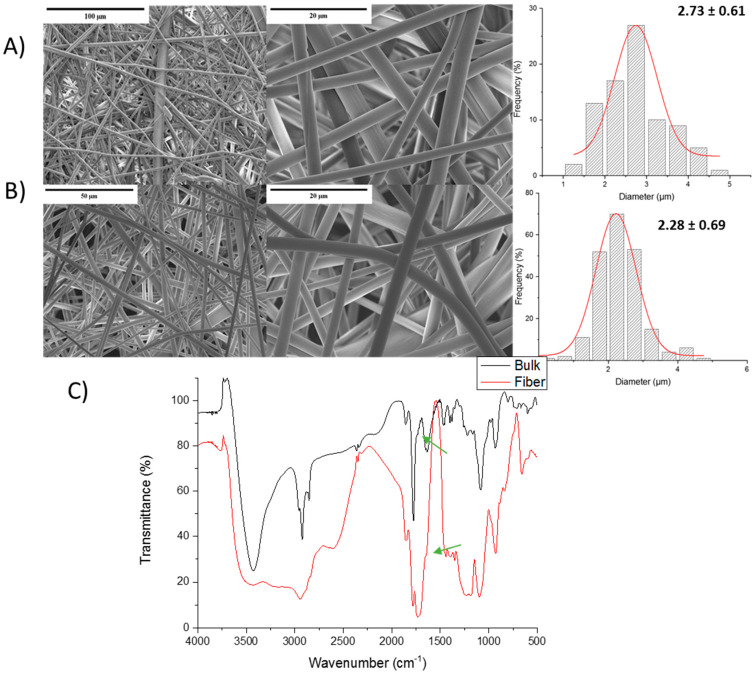
SEM images and fiber diameter of (**A**) the MVE-MA copolymer; and (**B**) the MVE-MA-PEG hydrogel. (**C**) FTIR spectra of the bulk and fiber hydrogels.

Finally, the MVE-MA-based fibers were evaluated (Figure 8). Taking into account the results obtained for the iBu-MA system, in this case, the role of the C–O bonds, being more hydrophilic and with greater mobility of the polymer chain than the iBu side groups, seem to facilitate the formation of fibers. These fibers are similar to those obtained for the Et-MA system, presenting a regular micro-size for both pure homopolymer (Figure 7A) and hydrogel (Figure 8B). The fiber diameters of both systems were quite similar, being slightly lower for hydrogel fiber, 2.73 ± 0.61 µm in comparison with pure copolymer 2.28 ± 0.69 µm. As it could be observed in the size distribution diagrams, the hydrogel presents a narrower size distribution; this could be ascribed to the crosslinking process that homogenized the fibers. It could also highlight that the hydrogel fiber mats do not show any fiber merge as a result of the crosslinking process, but they are less linear than copolymer fibers, showing certain curvature compared to the copolymer fiber, likely induced by the crosslinking. This result is a highly interesting point since the fibers do not lose their high surface as a consequence of the hydrogel forming process. The effective formation of this hydrogel was also confirmed by shoulders at 1730 cm^−1^ (green arrows) on the FITR spectra, Figure 8C.

## 4. Conclusions

In this work, ethylene-co-maleic anhydride, isobutylene-co-maleic anhydride and methyl vinyl ether-co-maleic anhydride hydrogels were synthesized by crosslinking with PEG in acetone/DMF solution. The crosslinking process was carried out by an esterification reaction between the hydroxyl groups of the polyethylene glycol and the carboxylic acid formed in the anhydride ring opening. The experimental results proved the successful crosslinking by the presence of the ester characteristic peaks on the FTIR spectra and some carboxylic acid moieties. The main properties of the hydrogels were highly dependent on the comonomer present. The maximum swelling behavior was achieved for methyl vinyl ether-substituted hydrogel, the reduction in the swelling capacity being ascribed to the hydrophobicity for ethylene comonomer and hydrophonicity and steric hindrance for isobutyl comonomer. The thermal stability and the mechanical properties were also affected by the properties of the comonomers, presenting methyl vinyl ether substituted hydrogel the higher thermal stability and being significantly tougher than isobutyl hydrogel, resulting in a maximum stress of 1.61 MPa in compression and the strain break at 95%. In addition, the iBu-MA-PEG presents the lower mechanical properties due to the lower mobility of the isobutylene groups that makes this hydrogel brittle. On the other hand, the fiber preparation by using electrospinning was evaluated in order to explore other possible applications of these materials in tissue engineering or wound healing applications. In this case, it could be noticed that the best candidate for these hydrogel fibers could be MVE-MA–PEG system. These fibers present highly regular fiber diameters, both of the copolymer and the hydrogel, compared to the other two systems. Overall, these materials present good mechanical properties, which it is a common drawback for hydrogels, in addition to a good ability to be manufactured as fiber mats by electrospinning, which could increase the potential use of these materials in drug delivery, wound healing and tissue engineering applications.

## Data Availability

The datasets generated during and/or analyzed during the current study are available from the corresponding author on reasonable request.
